# Supplementary material for: Structural ordering of the Plasmodium berghei circumsporozoite protein repeats by inhibitory antibody 3D11
Source: eLife. 2020 Nov 30;9:e59018. doi: 10.7554/eLife.59018 (PMC7704109; doi:10.7554/eLife.59018)
Supplement: Supplementary file 1. — (A) Hydrogen-bonding propensity for each simulated motif and lifetime of each β-turn for the four PfCSP-derived peptides. (B) Hydrogen-bonding propensity for each simulated motif and lifetime of each β-turn for the four PbCSP-derived peptides. [file elife-59018-supp1.docx]

**Supplementary File 1**

**A**

| **Peptide** | **KQPA** | | **NPDP** | | | **NVDP** | | | | **NPNA** | | | | |
| --- | --- | --- | --- | --- | --- | --- | --- | --- | --- | --- | --- | --- | --- | --- |
| **Motif** | **ADGN** | **DPNA** | **DPNA** | **NPNV** | **DPNA** | **DPNA** | **NPNV** | **DPNA** | **NPNV** | **NPNA** | **NPNA** | **NPNA** | **NPNA** | **NPNA** |
| **β-turn** | 0.34  ±0.04 | 0.38  ±0.03 | 0.37  ±0.06 | 0.17  ±0.04 | 0.38  ±0.05 | 0.43  ±0.04 | 0.17  ±0.05 | 0.44  ±0.07 | 0.16  ±0.02 | 0.30  ±0.03 | 0.24  ±0.05 | 0.42  ±0.04 | 0.33  ±0.05 | 0.19  ±0.03 |
| **β-turn Lifetime (ns)** | 3.53  ±0.30 | 3.94  ±0.26 | 4.22  ±0.44 | 2.79  ±0.27 | 3.97  ±0.26 | 4.31  ±0.27 | 3.20  ±0.32 | 5.52  ±0.55 | 2.27  ±0.16 | 3.80  ±0.24 | 4.72  ±0.38 | 4.35  ±0.26 | 4.26  ±0.25 | 2.21  ±0.09 |
| **Backbone-Backbone H-bonds** | 0.34  ±0.04 | 0.68  ±0.06 | 0.77  ±0.13 | 0.30  ±0.04 | 0.69  ±0.09 | 0.78  ±0.10 | 0.36  ±0.07 | 0.86  ±0.13 | 0.30  ±0.04 | 0.59  ±0.06 | 0.48  ±0.08 | 0.74  ±0.06 | 0.58  ±0.07 | 0.24  ±0.04 |
| **Pseudo**  **β-turn** | 0.00  ±0.00 | 0.07  ±0.02 | 0.10  ±0.02 | 0.04  ±0.01 | 0.08  ±0.01 | 0.10  ±0.02 | 0.02  ±0.01 | 0.10  ±0.02 | 0.02  ±0.01 | 0.09  ±0.03 | 0.03  ±0.01 | 0.09  ±0.04 | 0.06  ±0.03 | 0.06  ±0.01 |
| **Backbone-Sidechain H-bonds** | 0.02  ±0.01 | 0.15  ±0.03 | 0.22  ±0.05 | 0.15  ±0.03 | 0.18  ±0.03 | 0.21  ±0.04 | 0.11  ±0.04 | 0.19  ±0.04 | 0.13  ±0.04 | 0.27  ±0.07 | 0.07  ±0.02 | 0.22  ±0.07 | 0.16  ±0.07 | 0.21  ±0.04 |

**B**

| **Peptide** | **NPND** | | **NAND** | | **PAPP** | | **MIX** | |
| --- | --- | --- | --- | --- | --- | --- | --- | --- |
| **Motif** | **NPND** | **NPND** | **PPNA** | **PPNA** | **PPNA** | **PPNA** | **NPND** | **PPNA** |
| **β-turn** | 0.14  ±0.07 | 0.13  ±0.04 | 0.31  ±0.07 | 0.19  ±0.05 | 0.24  ±0.07 | 0.29  ±0.08 | 0.10  ±0.03 | 0.16  ±0.03 |
| **β-turn Lifetime (ns)** | 4.96  ±1.22 | 2.46  ±0.21 | 3.47  ±0.34 | 2.54  ±0.17 | 3.16  ±0.22 | 1.30  ±0.08 | 2.82  ±0.33 | 2.78  ±0.20 |
| **Backbone-BackboneH-bonds** | 0.14  ±0.07 | 0.13  ±0.04 | 0.41  ±0.06 | 0.28  ±0.06 | 0.32  ±0.08 | 0.40  ±0.08 | 0.10  ±0.03 | 0.25  ±0.03 |
| **Pseudo**  **β-turn** | 0.01  ±0.01 | 0.03  ±0.01 | 0.00  ±0.01 | 0.01  ±0.01 | 0.00  ±0.01 | 0.02  ±0.01 | 0.01  ±0.01 | 0.01  ±0.01 |
| **Backbone-Sidechain H-bonds** | 0.02  ±0.01 | 0.08  ±0.03 | 0.01  ±0.01 | 0.03  ±0.01 | 0.01  ±0.01 | 0.03  ±0.01 | 0.06  ±0.02 | 0.03  ±0.01 |
