# Supplementary material for: Structural ordering of the Plasmodium berghei circumsporozoite protein repeats by inhibitory antibody 3D11
Source: eLife. 2020 Nov 30;9:e59018. doi: 10.7554/eLife.59018 (PMC7704109; doi:10.7554/eLife.59018)
Supplement: Supplementary file 2. — Rows are shaded according to the number of times interactions are observed between all four crystal structures, summed in the final column. [file elife-59018-supp2.docx]

**Supplementary File 2**

|  | **Antigen** | | | | **Interaction** | **3D11-HC** | | **3D11-KC** | | **Total** |  |  |
| --- | --- | --- | --- | --- | --- | --- | --- | --- | --- | --- | --- | --- |
|  | **PAPP**  **(BSA Å^2^)** | **NAND**  **(BSA Å^2^)** | **NPND**  **(BSA Å^2^)** | **Mixed**  **(BSA Å^2^)** |  |  |  |  |  |  |  |  |
|  | **Pro1 (0)** | **Pro1 (30)** | **Pro1 (2)** |  |  |  | |  | |  |  |  |
|  | **Ala2 (0)** | **Pro2 (0)** | **Pro2 (7)** | **Pro2 (0)** |  |  | |  | |  |  |  |
|  | **Pro3 (8)** | **Pro3 (8)** | **Pro3 (7)** | **Pro3 (7)** |  |  | |  | |  |  |  |
|  | **Pro4 (104)** | **Pro4 (102)** | **Pro4 (107)** | **Pro4 (110)** |  |  | |  | |  |  |  |
|  | Pro | Pro | Pro | Pro | vdW | Tyr32, Ala95, Ala101, Tyr102 | | Arg46, Asp55 | | 4 |  |  |
|  |  | Pro |  | Pro | vdW | Tyr27 | |  | | 2 |  |  |
|  | Pro^O^ | Pro^O^ | Pro^O^ | Pro^O^ | HB | Tyr32^OH^ | |  | | 4 |  |  |
|  | **Asn5 (99)** | **Asn5 (104)** | **Asn5 (98)** | **Asn5 (94)** |  |  | |  | |  |  |  |
|  | Asn | Asn | Asn | Asn | vdW | Tyr32, Ala95 | | Arg46, Ser49, Leu50, Glu53 | | 4 |  |  |
|  |  |  | Asn | Asn | vdW | Ala101 | |  | | 2 |  |  |
|  | Asn^O^ | Asn^O^ | Asn^O^ | Asn^O^ | HB |  | | Arg46^NH1^, Arg46^NH2^ | | 4 |  |  |
|  | Asn^ND2^ | Asn^ND2^ | Asn^ND2^ | Asn^ND2^ | HB |  | | Ser49^OG^ | | 4 |  |  |
|  |  | Asn^ND2^ |  |  | HB |  | | Glu53^OE1^ | | 1 |  |  |
|  | **Ala6 (49)** | **Ala6 (48)** | **Pro6 (62)** | **Pro6 (65)** |  |  | |  | |  |  |  |
|  | Ala | Ala | Pro | Pro | vdW | Tyr32 | | Tyr32, Asn34, Arg46, Ser49, Leu50 | | 4 |  |  |
|  | **Asn7 (155)** | **Asn7 (155)** | **Asn7 (155)** | **Asn7 (155)** |  |  | |  | |  |  |  |
|  | Asn | Asn | Asn | Asn | vdW | His35, Ser94, Ala95 | | Tyr32, Asn34, Arg46, Trp89, Gly91 | | 4 |  |  |
|  |  |  | Asn |  | vdW |  | | Gln90 | | 1 |  |  |
|  | Asn^O^ | Asn^O^ | Asn^O^ | Asn^O^ | HB |  | | Asn34^ND2^ | | 4 |  |  |
|  | Asn^OD1^ | Asn^OD1^ | Asn^OD1^ | Asn^OD1^ | HB |  | | Arg46^NH1^ | | 4 |  |  |
|  | Asn^ND2^ | Asn^ND2^ | Asn^ND2^ | Asn^ND2^ | HB | Ala95^O^ | |  | | 4 |  |  |
|  | **Asp8 (98)** | **Asp8 (98)** | **Asp8 (98)** | **Asp8 (98)** |  |  | |  | |  |  |  |
|  | Asp | Asp | Asp | Asp | vdW | Asn33, His35, Tyr50 | | Tyr32, Gly91, Arg96 | | 4 |  |  |
|  | Asp | Asp |  | Asp | vdW |  | | Trp89 | | 3 |  |  |
|  | Asp^OD2^ | Asp^OD2^ | Asp^OD2^ | Asp^OD2^ | HB | Asn33^ND2^ | |  | | 4 |  |  |
|  | Asp^OD1^ | Asp^OD1^ | Asp^OD1^ | Asp^OD1^ | SB | His35^NE2^ | |  | | 4 |  |  |
|  | Asp^OD2^ | Asp^OD2^ | Asp^OD2^ | Asp^OD2^ | SB | His35^NE2^ | | Arg96^NE^, Arg96^NH2^ | | 4 |  |  |
|  | **Pro9 (106)** | **Pro9 (106)** | **Pro9 (98)** | **Pro9 (108)** |  |  | |  | |  |  |  |
|  | Pro | Pro | Pro | Pro | vdW | Asn33, Tyr50 | | Tyr27D, Tyr32, Gly91, Arg96 | | 4 |  |  |
|  | Pro | Pro |  | Pro | vdW |  | | Thr92 | | 3 |  |  |
|  | Pro |  | Pro | Pro | vdW |  | | Asp28 | | 3 |  |  |
|  | Pro^O^ | Pro^O^ | Pro^O^ | Pro^O^ | HB | Asn33^ND2^ | |  | | 4 |  |  |
|  | **Ala10 (36)** | **Pro10 (42)** | **Pro10 (45)** | **Ala10 (37)** |  |  | |  | |  |  |  |
|  | Ala | Pro | Pro | Ala | vdW | Asn33, Tyr50, Tyr52 | |  | | 4 |  |  |
|  | **Pro11 (96)** | **Pro11 (98)** | **Pro11 (99)** | **Pro11 (98)** |  |  | |  | |  |  |  |
|  | Pro | Pro | Pro | Pro | vdW | Asn33, Tyr50, Tyr52, Asn54, Val56, Asn58 | |  | | 4 |  |  |
|  |  |  | Pro^O^ |  | HB | Asn54^ND2^ | |  | | 1 |  |  |
|  | **Pro12 (11)** | **Pro12 (7)** | **Pro12 (8)** | **Pro12 (10)** |  |  | |  | | | |  |
|  | **Asn13 (36)** | **Asn13 (51)** | **Asn13 (21)** | **Asn13 (45)** |  | |  | |  | | |  |
|  |  |  | **Pro14 (36)** |  |  | |  | |  | | |  |
|  |  |  | **Asn15 (0)** |  |  | |  | |  | | |  |
| **H-bonds** | 9 | 10 | 10 | 9 |  | |  | |  | | |  |
| **Salt Bridges** | 4 | 4 | 4 | 4 |  | |  | |  | | |  |
| **Core epitope* BSA (Å^2^)** | 743 | 753 | 762 | 765 |  | |  | |  | | |  |
| **Total BSA (Å^2^)** | 798 | 849 | 843 | 827 |  | |  | |  | | |  |

* Core epitope consists of residues 4 to 11 in each peptide (PN(A/P)NDP(A/P)P)

vdW: van der Waals interaction (5.0 Å cut-off)

HB: hydrogen bond (3.8 Å cut-off)

SB: salt bridge (4.0 Å cut-off)
