# Supplementary material for: Structural ordering of the Plasmodium berghei circumsporozoite protein repeats by inhibitory antibody 3D11
Source: eLife. 2020 Nov 30;9:e59018. doi: 10.7554/eLife.59018 (PMC7704109; doi:10.7554/eLife.59018)
Supplement: Supplementary file 3. [file elife-59018-supp3.docx]

**Supplementary File 3**

|  | **Antigen (BSA Å^2^)** | | **Interaction** | **3D11-HC** | **3D11-KC** |
| --- | --- | --- | --- | --- | --- |
|  |  | **Pro25 (7)** | vdW |  | Ser56 |
|  |  | **Pro27 (12)** | vdW | Tyr32 |  |
|  |  | **Pro28 (99)** | vdW | Tyr32, Ala95, Ala101 |  |
|  |  | Pro^O^ | HB | Tyr32^OH^ |  |
|  |  | **Asn29 (105)** | vdW |  | Tyr32, Arg46, Ser49, Leu50, Glu53 |
|  |  | Asn^ND2^ | HB |  | Glu53^OE1^ |
|  |  | Asn^O^ | HB |  | Arg46^NH1^ |
|  |  | **Pro30 (71)** | vdW | Ala95 | Arg46 |
|  |  | **Asn31 (153)** | vdW |  | Asn34, Arg46, Trp89 |
|  |  |  | SB |  |  |
|  |  | Asn^ND2^ | HB | Ala95^O^ |  |
|  |  | Asn^O^ |  |  | Asn34^ND2^ |
|  |  | **Asp32 (98)** | vdW | Asn33, His35 | Asn34, Gly91, Arg96 |
|  |  | Asp^O^ | HB | Asn33^ND2^ |  |
|  |  | Asp^OD1^ | HB,SB | Asn33^ND2^ | Arg96^NH2^, Arg96^NE^ |
|  |  | Asp^OD2^ | HB,SB | His35^NE2^ |  |
|  |  | **Pro33 (103)** | vdW |  | Tyr27D, Tyr32, Gly91 |
|  |  | Pro^O^ | HB | Asn33^ND2^ |  |
|  |  | **Pro34 (41)** | vdW | Tyr52, Asn33 |  |
|  |  | **Pro35 (106)** | vdW | Tyr50, Tyr52, Asn58 |  |
|  |  | **Pro36 (13)** | vdW | Asn54, Lys56 |  |
|  |  | Pro^O^ | HB | Asn54^ND2^ |  |
|  |  | **Asn37 (13)** | vdW | Asn54 |  |
|  |  | Asn^OD1^ | HB | Asn54^ND2^ |  |
|  |  | **Ala38 (2)** | vdW | Asn54 |  |
| **H-bonds** |  | 13 |  |  |  |
| **Salt Bridges** |  | 4 |  |  |  |
| **Core epitope* BSA (Å^2^)** |  | 776 |  |  |  |
| **Total BSA (Å^2^)** |  | 823 |  |  |  |
|  |  |  |  |  |  |

*Core epitope consists of residues 28 to 35 in PbCSP (PNPNDPPP)

vdW: van der Waals interaction (5.0 Å cut-off)

HB: hydrogen bond (3.8 Å cut-off)

SB: salt bridge (4.0 Å cut-off)
